# Nature of antireflux barrier formed by Nissen fundoplication surgery

**DOI:** 10.1038/s41598-025-14104-x

**Published:** 2025-09-30

**Authors:** Robert Lee, Ravinder K. Mittal, Kenneth J. Chang, Ninh T. Nguyen

**Affiliations:** 1 Department of Medicine/Division of Gastroenterology, University of California Irvine Medical Center, Orange, CA USA; 2 Department of Medicine/Division of Gastroenterology, University of California San Diego, 9500 GillmanDrive, MC 0061, La Jolla, CA 92093-0990 USA; 3 Department of Surgery, University of California Irvine Medical Center, Orange, CA USA; 4 Department of Gastroenterology, Hoag Hospital, Irvine, CA USA

**Keywords:** Fundoplication, Antireflux barrier, Lower esophageal sphincter, Gastroesophageal reflux, Gastro-esophageal flap valve, Health care, Medical research

## Abstract

Nissen fundoplication is an effective surgical treatment for the treatment of gastroesophageal reflux disease but the exact mechanism by which it works remains debatable. We determined the effects of Nissen (360° wrap) and Toupet (270° wrap) fundoplication on the esophagogastric junction (EGJ) pressure in response to distension of the stomach in an ex-vivo preparation of the porcine esophagus and stomach. The duodenal opening was sealed, and stomach cannulated for gas insufflation using a barostat device. The effect of gastric distension on the EGJ was measured, before and after fundoplication, using high-resolution esophageal manometry impedance (HRMZ) catheter as well as with the functional luminal imaging probe (FLIP). Prior to fundoplication, insufflation of the stomach resulted in an immediate leakage of gas through the esophageal orifice with minimal gastric distension. Following fundoplication, the stomach distended like a balloon without leakage of air through the esophagus. The HRMZ recordings show an increase in the EGJ pressure with gastric distension that was 2–3 times higher than the increase in gastric pressure. The FLIP detected a graded decrease in the EGJ distensibility with gastric distension following fundoplication. We propose a novel mechanism by which fundoplication builds a mechanical barrier at the EGJ “sphincter like valve” to strengthen the anti-reflux barrier function.

## Introduction

The pathophysiology of Gastroesophageal Reflux Disease (GERD) includes diverse mechanisms, the unifying theme is the disruption of the anti-reflux barrier (ARB) at the esophagogastric junction (EGJ). The ARB in health consists of the smooth muscle lower esophageal sphincter (LES), crural diaphragm (CD) and flap valve mechanism at the EGJ^1–3^. The key motor events in the disruption of ARB are: (1) Transient Lower Esophageal sphincter relaxation (TLESR), a neurally mediated reflex initiated by gastric distension that induces brainstem and vagus nerve mediated relaxation of the LES and CD^2,4,5^. (2) An anatomical separation between the LES and crural diaphragm resulting in the formation of a sliding hiatus hernia, which impairs the LES and CD, function^6–8^. The hiatus hernia is also a two-step pressure pump of gastroesophageal reflux^9^; gastric contents move into the herniated stomach during the expiratory phase of respiratory cycle, and then from herniated stomach into the esophagus in the inspiratory phase, due to pressure gradients generated in the thorax and abdomen related to respiration and swallow-induced LES relaxation^9,10^. (3) Loss of gastroesophageal flap valve mechanism resulting from the sliding hiatus hernia is another possible factor^2,11^. The reduction of hiatus hernia is an important step in the anti-reflux surgery^12^. (4) Finally, hormonal factors such as endogenous secretin and Cholecystokinin (CCK) that are known to decrease the LES pressures may also play important role in the disruption of ARB^13,14^.

Fundoplication, surgical or endoscopic is an effective treatment of gastroesophageal reflux disease^12,15–18^. Several mechanisms by which fundoplication prevents GER have been suggested; (1) it increases the LES pressure^19^, (2) reduces the frequency of TLESRs and renders TLESRs incomplete^20, ^ (3) it impairs vagally mediated LES relaxation^21,22, ^ (4) it augments the impact of gastrin in increasing LES pressure^23^. Studies show that fundoplication performed in cadavers and in an ex-vivo preparation of the harvested specimen of esophagus and stomach increases the EGJ pressure in response to gastric distension, which suggests a mechanical effect of fundoplication on the ARB^24–26^. The goal of our study was to re-examine the mechanical effect of partial and complete fundoplication on the EGJ pressure and GER in response to gastric distension using the “state of the art” manometry and reflux monitoring method, i.e., high resolution esophageal manometry impedance (HRMZ), and functional luminal imaging probe (FLIP). Secondary objectives included comparing the impact of loose vs. tight Nissen fundoplication on the EGJ response to gastric distension. Based on our studies, we hypothesize a novel mechanism by which Nissen fundoplication forms a mechanical ARB to prevent the GER.

## Methods

Studies were conducted in the specimens obtained from 15 pigs through EndoSim LLC (Hudson, Mass). The entire length of the esophagus, stomach and proximal duodenum was harvested by EndoSim personnel, in accordance with the animal ethics and guidelines committee. We received an exempt status from the IACUC of the University of California Irvine because these experiments were not on live tissues. Authors confirm that all methods are reported in accordance with the ARRIVE guidelines.

**Fig. 1:**
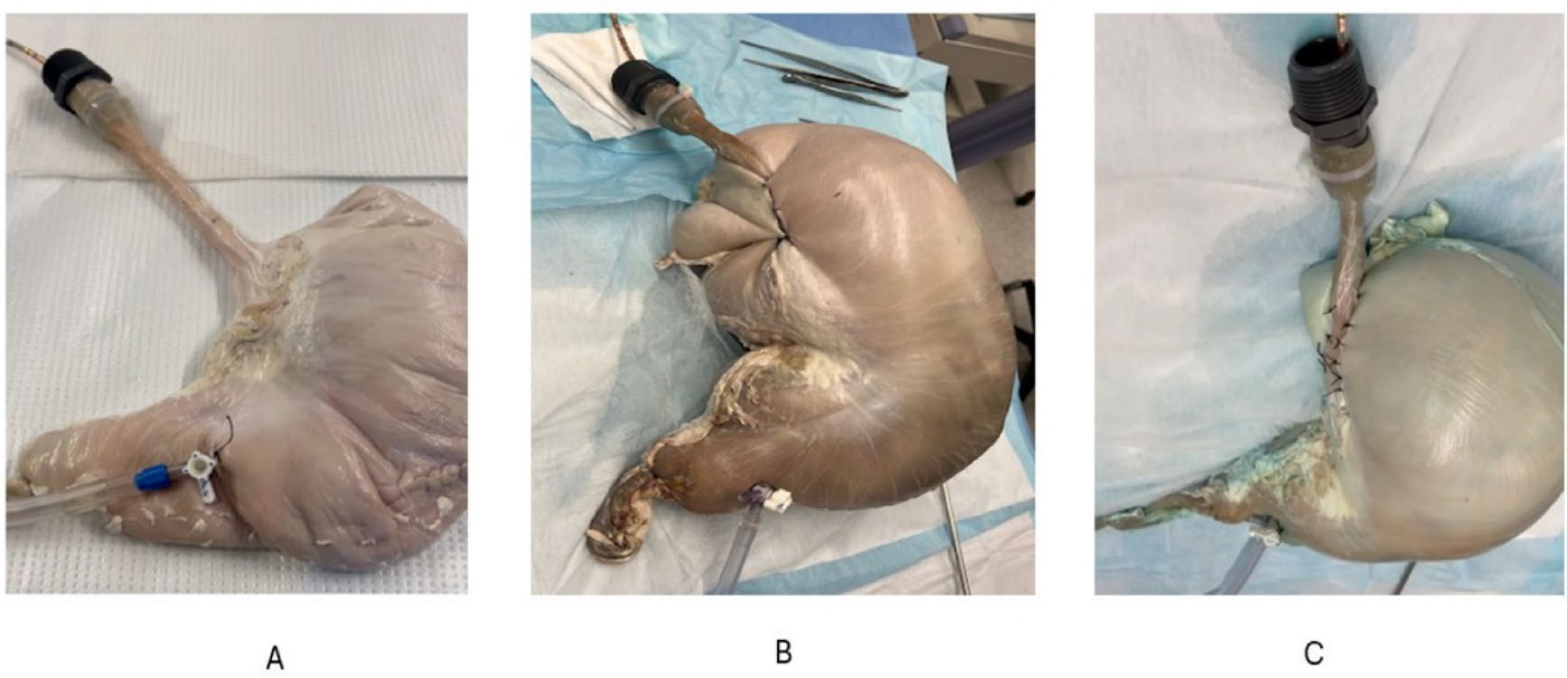
Gross appearance of porcine specimens. (**A**) Esophagus and stomach without fundoplication, duodenal opening is ligated close, and stomach is cannulated for air or saline infusion. A 36-channel HREMZ catheter is placed in the lumen of esophagus and stomach. (**B**) setup after 360° Nissen Fundoplication. (**C**) A 270° Toupet, partial fundoplication. Note that unlike before fundoplication, the stomach distends like a balloon following complete and partial fundoplication, suggesting a mechanical effect at the esophagogastric junction.

**Fig. 2:**
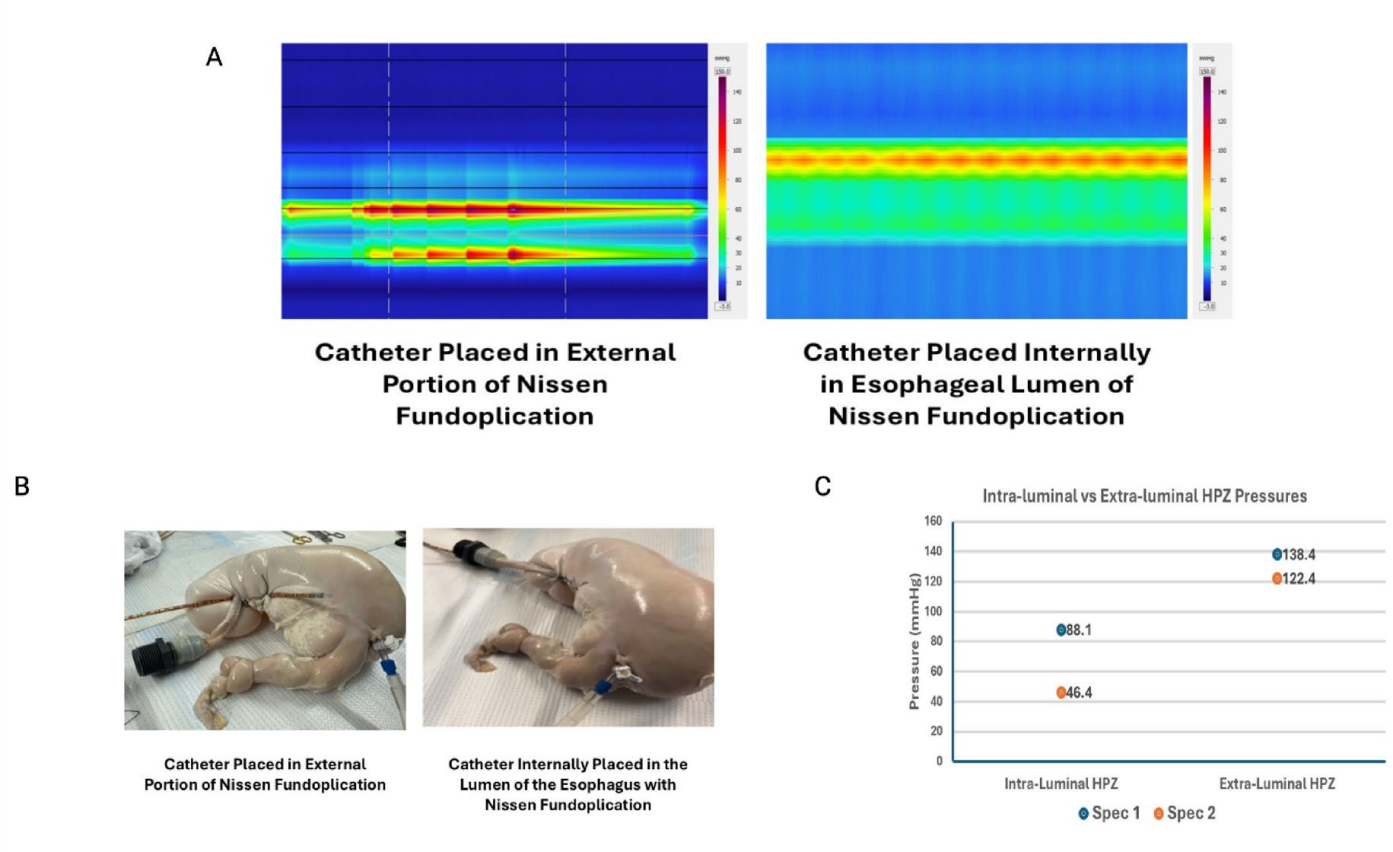
Experiments performed on 2 specimens with Nissen Fundoplication. (**A**) Pressure contour plots of the EGJ (black arrows) with the HREMZ catheter placed in the lumen of esophagus, and outside the lumen but inside the fundoplication wrap. The stomach was distended to 5, 7 and 10 mmHg. Gastric distension resulted in increase in pressure in both situations. (**B**) HRMZ catheter located in the extraluminal and intra-luminal locations. (**C**) The EGJ pressure from the extraluminal and intraluminal locations with stomach distended to 10 mmHg.

**Fig. 3:**
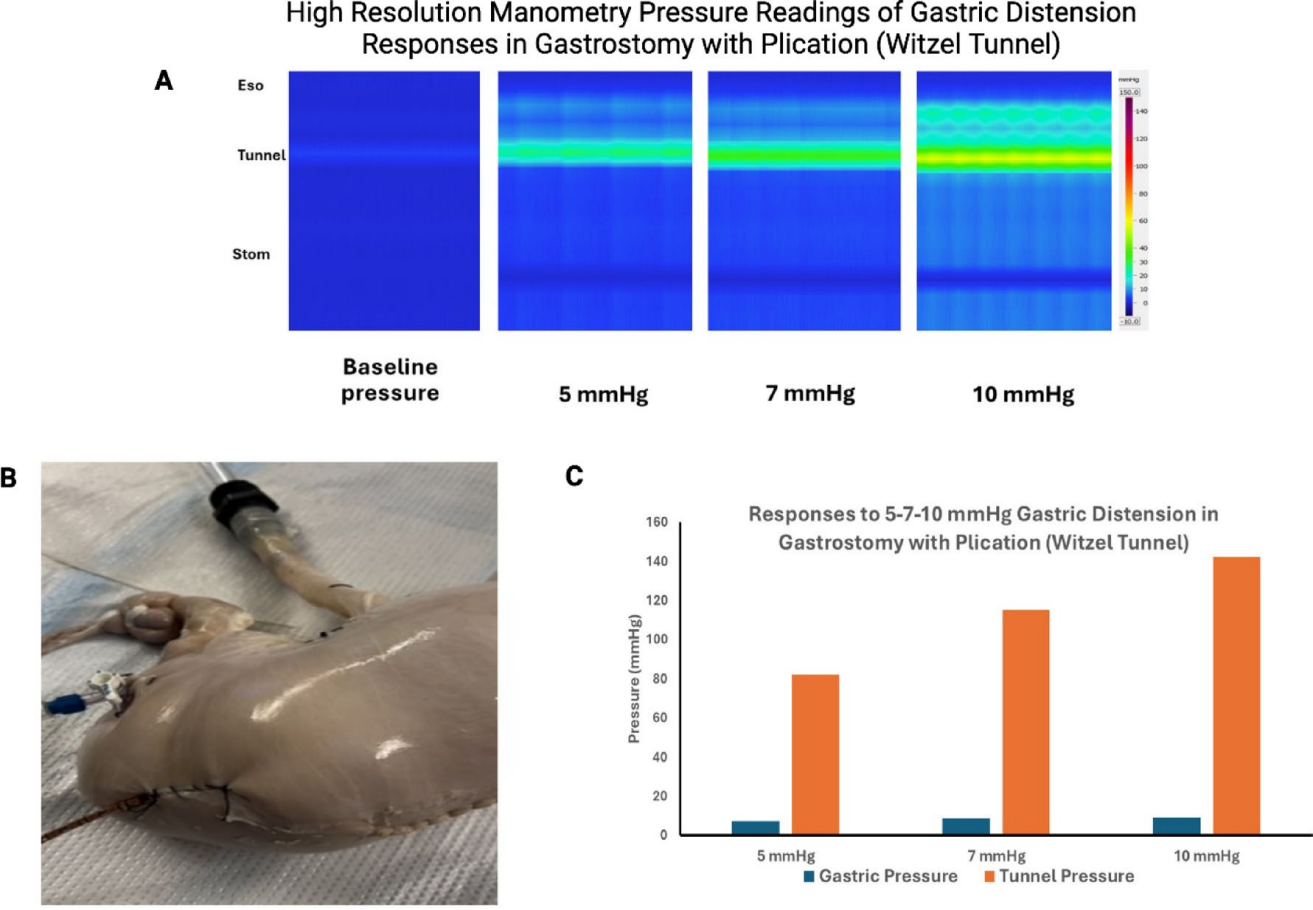
“Witzel Valve” created by a small gastrostomy with tunnel built by suturing the stomach wall over the catheter. (**A**) Pressure reading inside the tunnel and stomach during gastric distension to 5, 7 and 10 mmHg. (**B**) Visual appearance of “Witzel Valve with HREMZ catheter in place. (**C**) Gastric and tunnel pressure readings with gastric distension. Tunnel pressures showed a stepwise increase that was significantly higher than gastric pressures.

The duodenal opening was ligated close, and stomach was cannulated with a 3-way stopcock. Gastric distention was performed with (1) gas insufflation with carbon dioxide using an electronic endoflator (Karl Storz, El Segundo, CA), which works like a barostat, and (2) gastric infusion using an intravenous bag of saline hung high on a pole. The effect of gastric distension on the EGJ function was measured using 2 techniques, HRMZ and functional luminal imaging probe (FLIP). A 36-channel high-resolution esophageal manometry-impedance (HREMZ) catheter (Medtronic Inc, MN) was placed into the esophagus and stomach to capture pressure data and reflux episodes, measured as retrograde flow on the impedance measurements. The recordings were performed, before and during gastric distension with gas and saline under following conditions, (1) baseline condition, i.e., prior to fundoplication (Fig. 1A), (2) following complete fundoplication (Nissen procedure, Fig. 1B), (3) following partial fundoplication (Toupet procedure, Fig. 1C). The complete fundoplication (360° wrap) was performed by wrapping the gastric fundus around the entire circumference of the esophagus using three interrupted sutures. The partial or a 270° fundoplication was performed by wrapping gastric fundus around distal esophagus with an approximate 90° bare circumference of the esophagus. Three rows of sutures were placed, at approximately 10°’ clock and 2°’ clock positions. For the FLIP studies the probe (325 N, Medtronic Inc) was placed straddling across the EGJ. The FLIP bag was inflated with 60 ml saline. Gastric distension was performed with CO_2_ gas using the endoflator at the desired pressure dialed into the machine, prior to the onset of distension. HRMZ and FLIP recordings were obtained with gastric distension pressures of 0, 5, 7 and 10 mmHg. Gastric distensions with saline were performed to determine the volume of fluid at which reflux into the esophagus occurred first during the HRMZ recordings.

To better characterize the mechanical effect of fundoplication on the EGJ during gastric distension, 3 additional experiments were performed using the HRMZ probe. (1) In 2 specimens, the HREMZ catheter was placed between the fundoplication wrap and esophagus (instead of the esophagus lumen) (Fig. 2A–B). The pressure was recorded inside the wrap at 10 mmHg of gastric distension and compared with the EGJ pressure readings with the catheter placed inside the esophageal lumen. (2) In three specimens, the effects of a loose versus tight fundoplication on the EGJ pressure were studied. For the loose wrap, the circumferential length of the fundus used for the wrap was approximately 30% longer than for the tight fundoplication. (3) In one specimen we tested whether Nissen fundoplication works like a “Witzel valve”, a surgery often used to prevent leakage around the enterostomy tubes^27^. To test the above, the esophageal opening was ligated close at its proximal end. The HREMZ catheter was placed into the stomach through a small gastrostomy opening created surgically. A part of the HRMZ catheter proximal to the gastrostomy opening was buried in a tunnel built by suturing the stomach wall on the 2 sides of the catheter over the catheter (Fig. 3B). In the above set up, 4–5 pressure transducers were located in the stomach, another 4–5 pressure transducers in the tunnel, and the remainder outside the stomach. Recordings were obtained, before and during gastric distensions, with barostat pressure at 5, 7 and 10 mmHg.

### Data analysis

The HRMZ data were analyzed using the Manoview Eso 3.3 software (Medtronic, Minneapolis, MN USA). Measurement of pressure at the EGJ (inside the fundoplication wrap) was obtained using the e-sleeve function, 10 s prior to, and then during gastric distension, with gas and liquid. The manometric location of EGJ within the wrap was confirmed by manually pressing the catheter with a fingertip over the area of wrap. The volume of saline at which reflux into the esophagus, as detected by the impedance recording, was recorded. The FLIP measurements of the bag pressure, minimal bag diameter inside the fundoplication and EGJ distensibility were recorded at the gastric pressure of 0, 5, 7 and 10 mmHg, before and after fundoplication.

### Statistical methods

Comparisons of pressures and FLIP measurements before fundoplication, with partial fundoplication and Nissen fundoplication were assessed using ANOVA for repeat measures when distributions were normal and Friedman’s rank test when they followed a non-parametric pattern. Comparisons of saline volume thresholds to induce reflux were compared in a similar fashion. The 3-way comparisons were calculated using the Dunn-Bonferonni corrections. Data derived from the tight vs. loose fundoplication and gastrostomy experiments are described as descriptive observations.

## Results

### Complete fundoplication

**Fig. 4:**
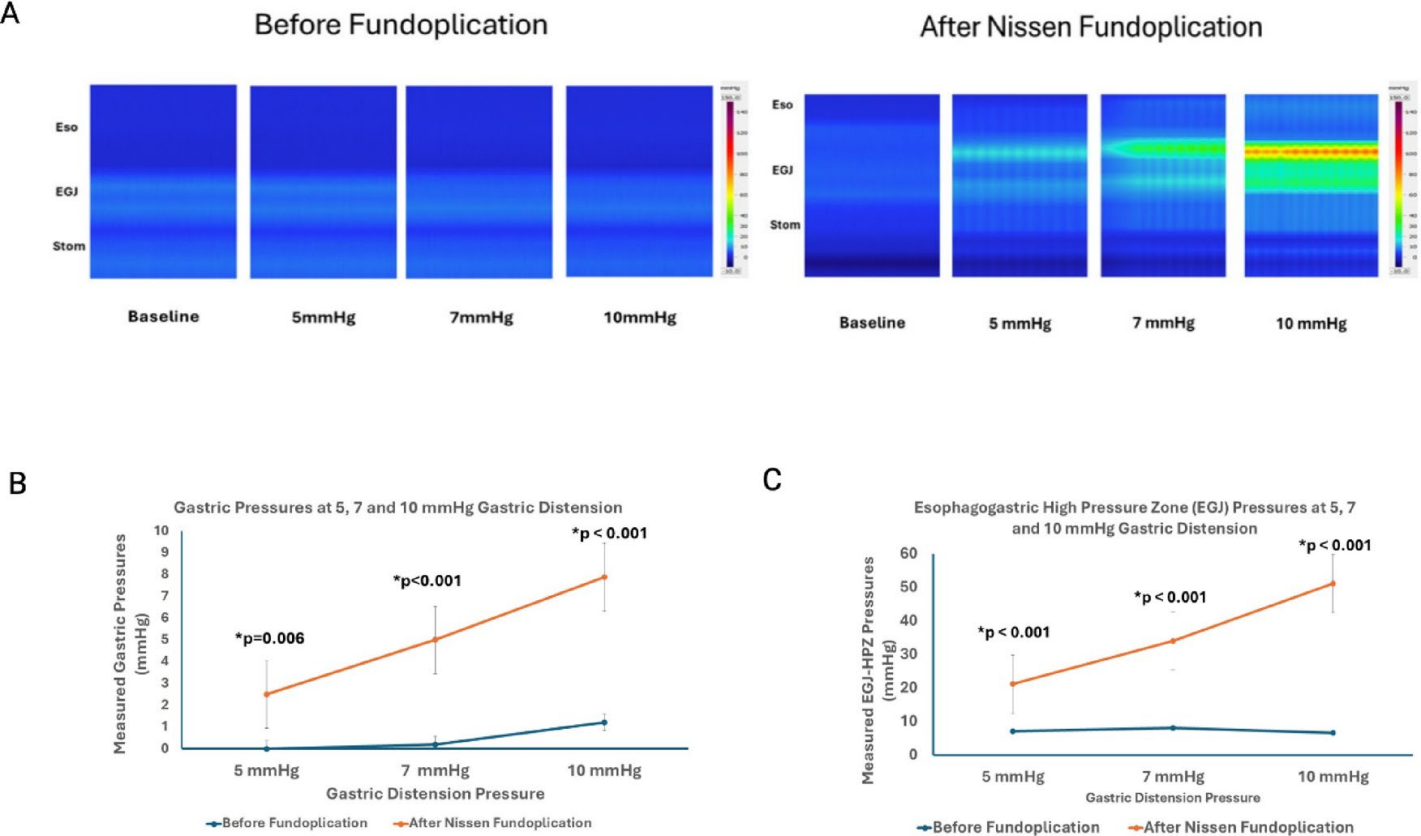
(**A**) Pressure recordings in the Esophagus (Eso), Esophagogastric Junction (EGJ) and Stomach (Stom) obtained prior to and then with gastric distension at 5, 7 and 10 mmHg pressure, before and after Nissen fundoplication. Gastric Pressure (**B**) and EGJ pressure (**C**) with gastric distension were higher following Nissen compared to before Nissen fundoplication.

Distension of stomach before fundoplication resulted in an immediate leakage of air into the esophagus. Visually, minimal inflation of the stomach was observed, irrespective of the pressure set in the barostat, i.e., 5, 7 and 10mmHg. Furthermore, the gastric pressure, as recorded by the HRMEZ catheter did not increase with the rise in the barostat pressure (Fig. 4A and B). Following fundoplication, before gastric distension, there was a small (2.8 mmHg) increase in the EGJ pressure (*p* = 0.002) but no change in gastric pressure (*p* = 0.41) (Fig. 4A—baseline), compared to before fundoplication. With gastric distension, however, the stomach distended like a balloon and there was no leakage of air from the esophagus (Fig. 1B). The gastric and EGJ pressure increased in a linear fashion with the increase in the barostat pressure (Fig. 4B); the increase in EGJ pressure with gastric distension was significantly higher than the increase in gastric pressure, (*p* < 0.001) (Fig. 4C). At 10mmHg barostat pressure, the gastric and EGJ pressure were 7.9 and 51.1 mmHg, respectively (Fig. 4B–C).

### Partial fundoplication

**Fig. 5:**
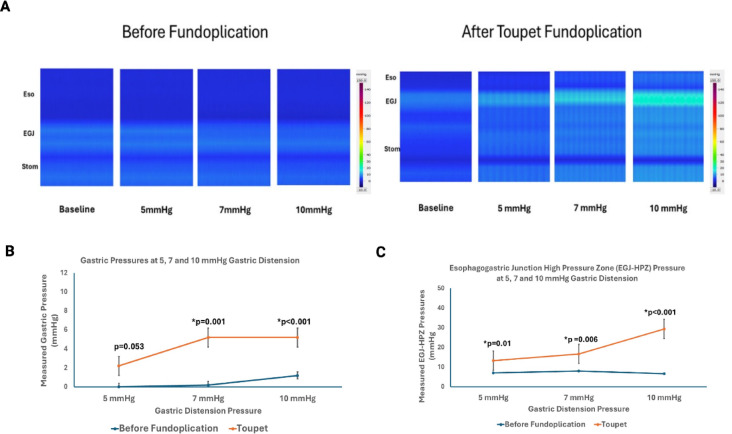
(**A**) Pressure recordings in the Esophagus (Eso), Esophagogastric Junction (EGJ) and Stomach (Stom), prior to, and with gastric distension at 5, 7 and 10 mmHg pressure, before and after partial (Toupet) fundoplication. Gastric Pressure (**B**) and EGJ pressure (**C**) with gastric distension were higher following partial fundoplication compared to before Toupet fundoplication.

**Fig. 6:**
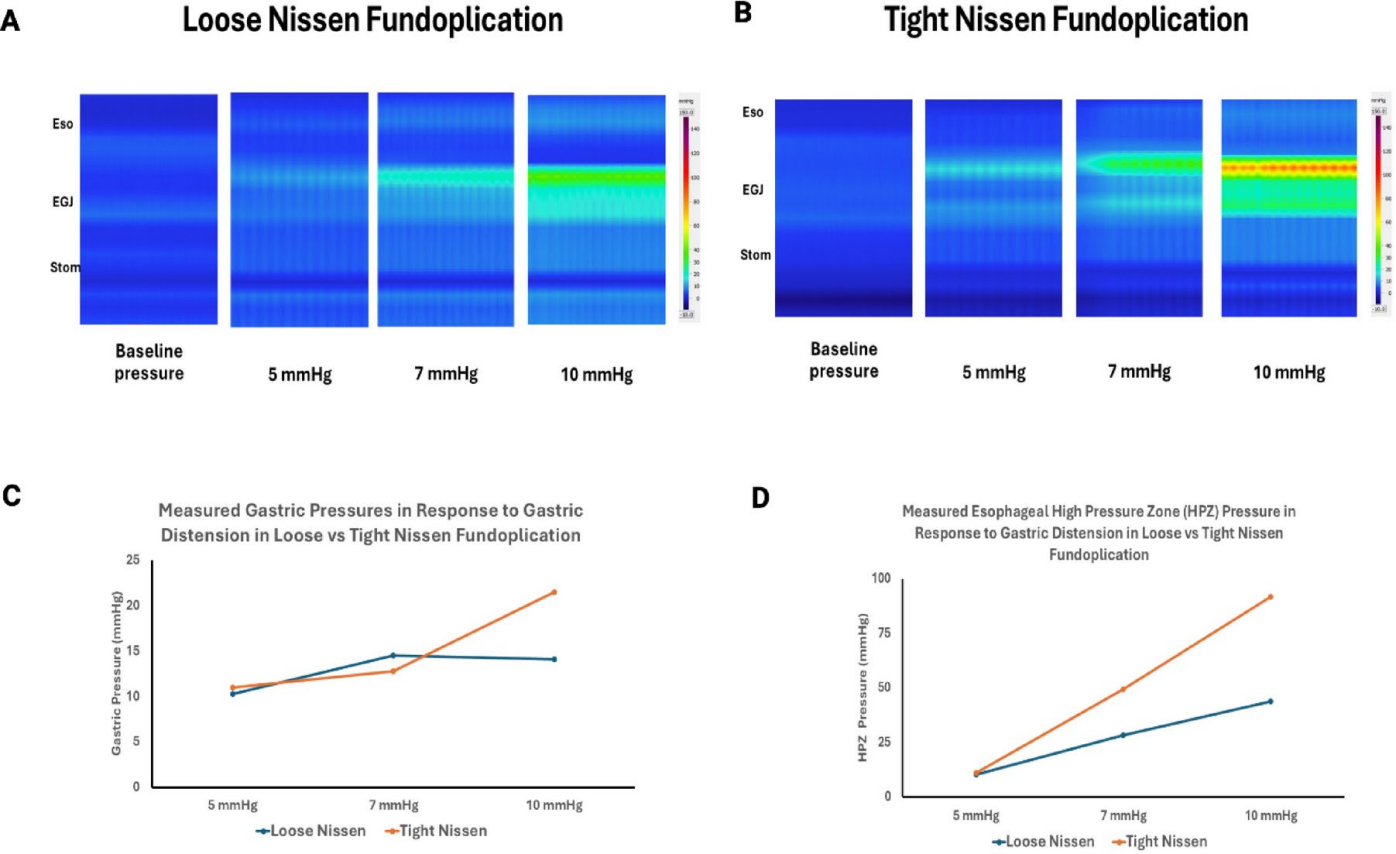
Gastric and EGJ pressure with loose (**A**) vs. tight (**B**) Nissen fundoplication. The gastric pressures during gastric distension with 5, 7 and 10 mmHg were similar between groups (**C**). The EGJ pressure were greater but not statistically significant with tight vs. loose fundoplication (**D**), likely related to small sample size.

Partial fundoplication also resulted in findings similar to complete fundoplication. Prior to distension, there was a statistically significant but very small (6–9 mmHg) increase in the EGJ pressure, compared to before fundoplication. (*p* < 0.001) and no change in gastric pressures (0.1) (Fig. 5A). With gastric distension, the stomach distended like a balloon (Fig. 1C). The gastric pressure was higher compared to before partial fundoplication (Fig. 5B), at all levels of gastric distensions. The EGJ pressure augmentation in response to gastric distension also occurred in a linear fashion (Fig. 6C), and the highest pressure was seen at 10 mmHg (Fig. 5C). The EGJ pressure was always higher than the gastric pressure, at all levels of distension, (Fig. 5B–C). The EGJ pressure at 10 mmHg gastric distension was significantly lower for partial fundoplication as compared to complete fundoplication, (35.4 vs. 60.2 mmHg, *p* < 0.001).

### HREM probe location: intraluminal vs. extra-luminal

When the HRMZ probe was located inside the fundoplication wrap but outside the esophageal lumen, there were no pressure sensors in the stomach (Fig. 2A). The gastric distension resulted in a large increase in the pressure recorded by the transducers located in the wrap, significantly higher than the pressure set up in the barostat (Fig. 2B) The pressure recorded by the probe in the extra-luminal location were higher than when the probe was located inside the lumen of esophagus (Fig. 2C).

### Loose vs. tight fundoplication

Following fundoplication, and prior to gastric distension, there was no difference in the EGJ pressure between loose vs. tight fundoplication groups (8 vs. 10.7 mmHg, *p* = 0.109). With gastric distension, the gastric pressures were similar in both groups. However, the increase in the EGJ pressure with gastric distension was higher in the tight fundoplication specimens as compared to the loose fundoplication (Fig. 6A–D).

### Distension of stomach with saline

**Fig. 7:**
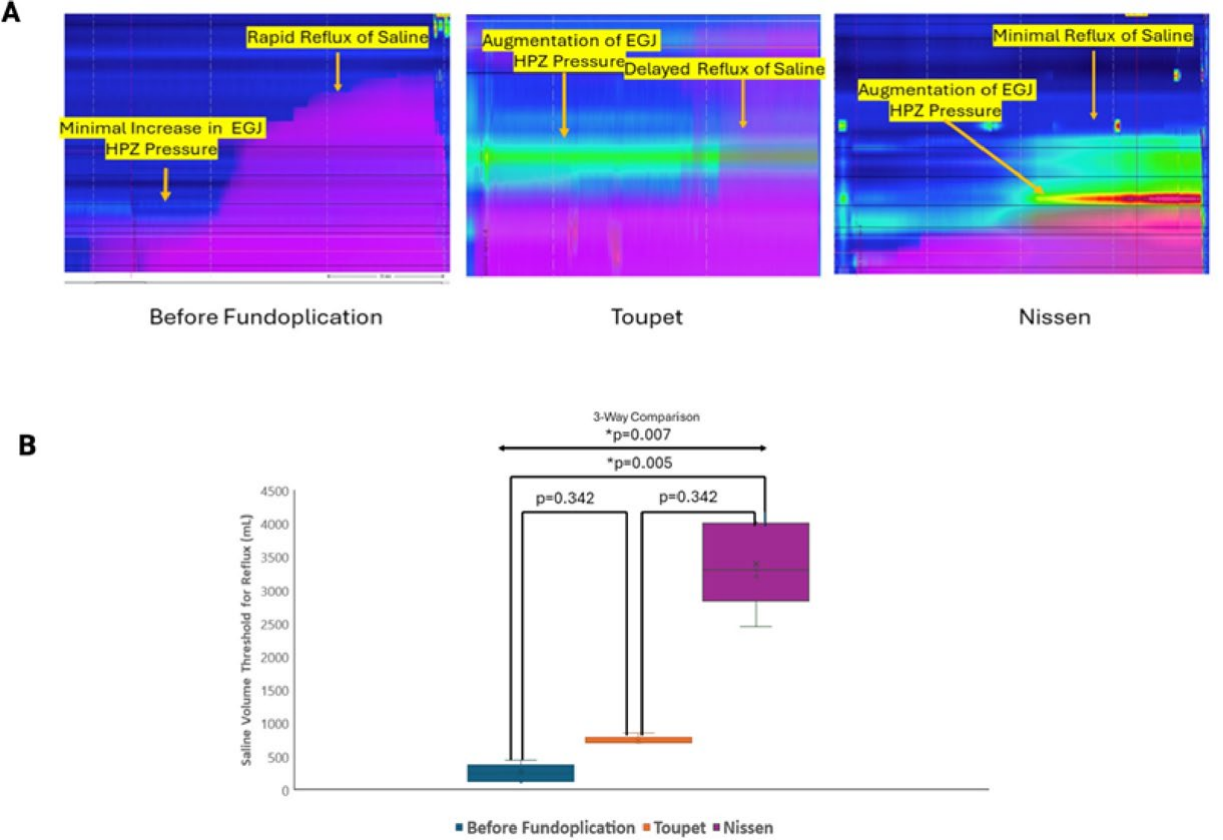
HREMZ pressure and impedance recordings during gastric distension with saline, at baseline, following Nissen and following Toupet fundoplication (**A**). Note, minimal changes in the EGJ pressure with rapid reflux of saline (purple) into esophagus at baseline. In contrast, note the augmentation of EGJ pressure during gastric distension, and delayed reflux of saline seen following Toupet and Nissen fundoplication. (**B**) Saline volume at the onset of reflux was higher following Nissen and Toupet fundoplication as compared to before fundoplication.

Prior to fundoplication, the saline infused into the stomach refluxed quickly into the esophagus at very low volume of saline. Following fundoplication, a significantly larger volume of saline was required at which reflux occurred into the esophagus (as seen visually and by impedance recording) (Fig. 7A). The volume of saline to induce reflux was higher in the complete as compared to partial fundoplication group (Fig. 7B).

### Gastrostomy with plication over HREM catheter (Witzel tunnel)

In the absence of gastric distension, the pressures recorded by all pressure channels, i.e., in the stomach, in the tunnel formed by the plication, and atmospheric pressure were similar. With gas insufflation of the stomach, the gastric pressure increased in a linear fashion with the increase the barostat pressure (Fig. 3C). The stomach distended like a balloon and there was no leakage of air from the gastrostomy opening (Fig. 3B). Most interestingly, the pressure transducers located in the tunnel formed by gastric wall plication showed a graded increase in pressure, which was significantly greater than the gastric pressure recorded by transducers located in the stomach, (Fig. 3A–C).

### FLIP measurements

**Fig. 8:**
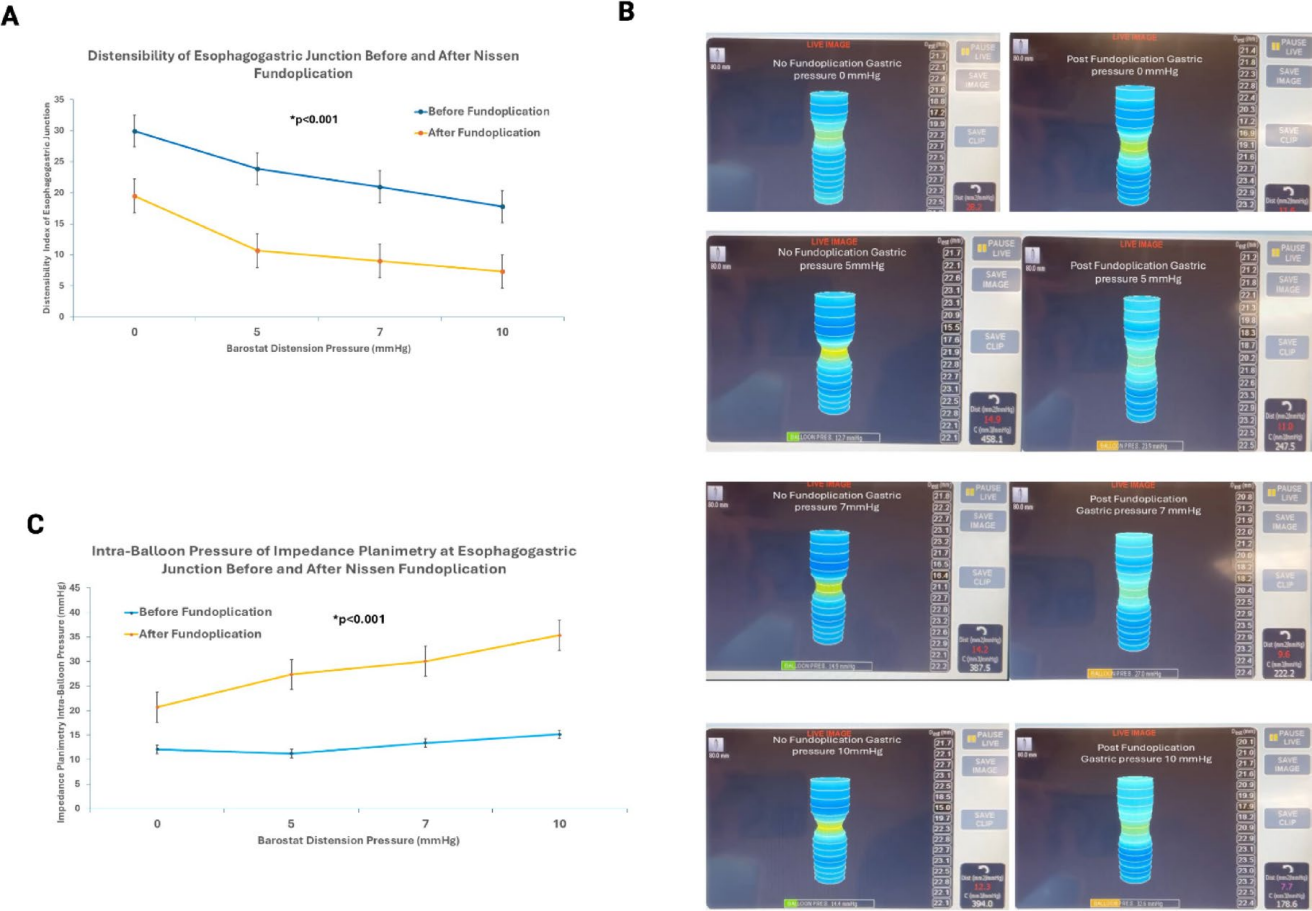
Functional luminal Imaging Probe Measurement before and after Nissen Fundoplication: Prior to fundoplication, gastric distension resulted in a dose depended increase in the bag pressure and decrease in the esophagogastric junction distensibility. Following Nissen fundoplication the increase in bag pressure and decrease in distensibility were significantly greater as compared to before fundoplication. A = mean data of distensibility before and after fundoplication, C= mean data of bag pressure before and after fundoplication, and c= images of the functional luminal imaging bag before and after fundoplication at different gastric distension pressures.

Prior to fundoplication, the inflated bag of the FLIP probe resulted in partial occlusion of the EGJ opening that resulted in distension of stomach with insufflation of gas into the stomach. Gastric distension resulted in a dose depended but small increase in the bag pressure and decrease in the EGJ distensibility before fundoplication. Following fundoplication, the increase in bag pressure, and decrease in the EGJ distensibility were significantly greater as compared to before fundoplication, (*p* < 0.05), (Fig. 8).

## Discussion

Our data show the following: (1) partial and complete fundoplication results in a mechanical barrier to the flow of stomach contents into the esophagus. (2) The increase in EGJ pressure in response to gastric distension, following fundoplication is larger with the 360° wrap as compared to 270° wrap. (3) The increase in EGJ pressure with gastric distension is significantly greater with tight, as compared to a loose fundoplication, (4) The EGJ response to gastric distension following fundoplication is similar to what is seen in the gastrostomy with plication of the gastric wall over the catheter, also known as the valve of Witzel, and finally, (5) The FLIP study showed reduction in the EGJ distensibility with gastric distension, in a dose dependent manner, following Nissen fundoplication.

The fact that the stomach distends like a balloon following fundoplication raised the possibility that fundoplication resulted in an esophageal luminal narrowing/stricture causing obstruction to the flow of stomach contents into the esophagus. However, such is not the case because fundoplication produced very small increases in the EGJ pressure compared to baseline state, prior to gastric distension. Furthermore, there is a graded increase in the EGJ pressure with graded distension of stomach, almost like a “dose-response curve”. Farrell et al. used harvested esophagus and stomach from the human cadavers and conducted ex-vivo experiments, similar to our pig model^24^. They used infusion manometry to measure the EGJ pressure and found that fundoplication resists GER, independent of the in-vivo anatomic relationships. Farrell also found that during gastric distension the LES pressure was 2 to 2.5 times higher than the gastric pressure^24^. We found even higher EGJ pressure than reported by Farrell et al. In their experiments, fundoplication was performed over a 60 F bougie, which was not the case in our study. We found that the increase in EGJ/LES pressure with gastric distension following fundoplication is dependent upon the tightness of the wrap. It is likely that placement of a bougie in the esophagus results in a looser wrap than without a bougie, and hence lower EGJ pressure in response to gastric distension. Michael et al. studied the angle of His and the esophagogastric area in an ex-vivo preparation of the porcine esophagus and stomach, before and after partial and complete fundoplication in response to gastric distension^26^. They found changes in the angle of HIS, reduction in the EGJ orifice size and reduction in gastroesophageal reflux following fundoplication. However, they did not record EGJ pressure in response to gastric distension. Hill et al. studied cadavers in-vivo with no hiatal hernia and found a gradient (defined as the gastric pressure at which flow occurs into the esophagus) across the EGJ in nearly all cadavers, and this gradient was increased by surgically accentuating the valve without a concomitant rise in pressure in the high-pressure zone^25^. Reduction of the hiatal hernia in cadaver and anchoring of the EGJ to a normal attachment, i.e., preaortic fascia, restored the valve, as seen through a gastrostomy. It also restored the pressure gradient between the esophagus and stomach. It appears that the restoration of valve technique of Hill is different from the commonly performed fundoplication technique and hence we can’t be sure of the reason for the gastroesophageal pressure gradient and accentuation of the gastroesophageal gradient following restoration of the “flap valve”. Our study is the first to record changes in the EGJ distensibility following fundoplication using FLIP. A decrease in the EGJ distensibility in response to gastric distension is consistent with the manometry findings of increase in the EGJ pressure. Patients with GERD have higher EGJ distensibility as compared to normals^28,29^. Reduction in the EGJ distensibility following fundoplication suggests an improvement in the EGJ valve competence.

Nissen described a case of chronic bleeding ulcer in the terminal esophagus and cardia^30,31^. Nissen resected the terminal esophagus including the cardia, performed a gastropexy and wrapped the two lateral folds of the stomach over the esophago-gastric anastomosis, similar to what he called the “valve of Witzel”. Nissen observed that in spite of the lack of cardia/LES, the patient had no symptoms of esophagitis in the long-term follow-up. Surgeons often perform a Witzel procedure to prevent leakage around the catheter of the enterostomy. The precise mechanism, by which Witzel procedure prevents leakage, is not known, to the best of our knowledge. We studied the effects of a Witzel valve by placing the manometry catheter through the gastrostomy and then wrapping the stomach wall over the manometry catheter. Distension of the stomach resulted in an increase in the pressure on the part of the catheter buried in the tunnel, similar to fundoplication. The increase in pressure occurred in a graded fashion and it was significantly greater than the increase in gastric pressure. Furthermore, no air leaked around the gastrostomy site, similar to how fundoplication around the EGJ prevents leakage of stomach contents into the esophagus.

**Fig. 9:**
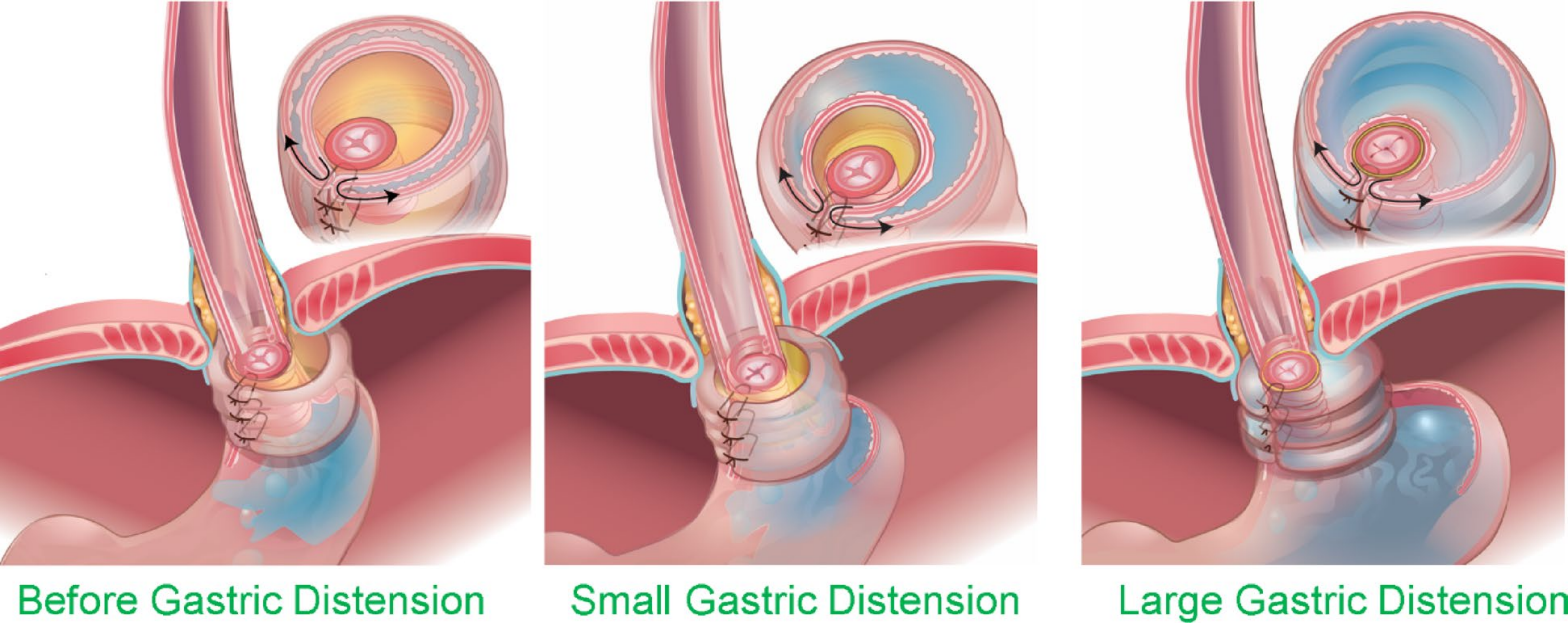
Schematic of the mechanism of the EGJ pressure increase following fundoplication. With gastric distension, there is an increase in the diameter of the main stomach but a decrease in diameter of the stomach wrapped around the esophagus, which constricts the esophagus to prevent gastroesophageal reflux.

With regards to the mechanism by which Nissen fundoplication increases the competency of the antireflux barrier, Farrell et al. suggested that with gastric distension, sutures between the 2 side of the fundus prevent the fundoplication from opening anteriorly^24^. They propose “since all tangential forces are balanced, radial forces provide circumferential esophageal occlusion”. We provide a simple alternative explanation for the mechanical effects of fundoplication. Fundoplication builds a unique number of “8 like” construct of the stomach around the distal esophagus (Fig. 9). In a cross-sectional view of the fundoplication architecture one can see how distension/expansion of the main part of the stomach increases its diameter at the expense of wrapped fundus around the esophagus, which resulting in a constriction around the esophagus. Greater the expansion of the main part of stomach, smaller the diameter of the wrapped fundus around the esophagus, thus explaining the “dose response like” effects of gastric distension on the EGJ pressure. To test the above hypothesis, we placed the manometry catheter between the wrap and esophagus (instead of esophageal lumen) and found that the gastric distension resulted in large increase in the pressure in the wrap zone, similar to what is seen when the catheter is placed in the esophageal lumen. The anatomy of the “Witzel valve” around the gastrostomy site in our study is indeed similar to fundoplication, and so is the effect of gastric distension in preventing leakage from the gastric opening.

Bardini using intraoperative high-resolution manometry observed increase in the LES pressure in response to gastric distension following fundoplication in humans^32^. However, the change in LES pressure observed in their study was relatively small. Furthermore, gastric distension in their study was not standardized, unlike our study where we were able to dial in the required gastric pressure using electronic endoflator. We describe exclusively the mechanical effect of the architecture created by fundoplication in our study. On the other hand, in the in-vivo human situation, with the live antireflux barrier (LES, crural diaphragm and flap valve) under the influence of myogenic, hormonal and neural responses, additional mechanisms might also be in play. Further studies are needed in humans, similar to the ones conducted in the current paper, to determine whether the EGJ pressure response to gastric distension is similar to what we observed in our current studies. We suggest that a standardized response to gastric distension induced increase in the esophago-gastric junction pressure at the time of surgery (in operation room) could be an important strategy to standardize Nissen fundoplication which we know works but at times lead to side effects. We found that the circumference of fundus used for wrap and the circumference of esophagus wrapped are two important determinants of the esophagogastric response to gastric distension. While surgeons focus on the circumference of esophagus that is wrapped, i.e., 360°, 270° etc. the circumference of fundus used for wrap is not generally paid attention to. We believe future studies should standardize both. If the above were to be true in live humans, one might be able to design a better Nissen procedure with less side effects, such as dysphagia, difficulty belching and gas bloat symptoms.

## Data Availability

All data pertaining to this manuscript are with Drs Ravinder K. Mittal (UCSD) and Robert Lee (UCI) and is available for the appropriate interested parties.
